# Supplementary material for: Spatially interacting phosphorylation sites and mutations in cancer
Source: Nat Commun. 2021 Apr 19;12:2313. doi: 10.1038/s41467-021-22481-w (PMC8055881; doi:10.1038/s41467-021-22481-w)
Supplement: Supplementary file 3 — Description of Additional Supplementary Files [file 41467_2021_22481_MOESM3_ESM.pdf]

Description of Additional Supplementary Files

File Name: Supplementary Data 1.
Description: Co-clustering phosphosites and cancer mutations in hybrid clusters.

File Name: Supplementary Data 2.
Description: Mutations found only by phosphosite-mutation co-clustering but not by mutation-only clustering.

File Name: Supplementary Data 3.
Description: Pathway enrichment analyses of proteins harboring hybrid clusters.

File Name: Supplementary Data 4.
Description: Protein domain enrichment analyses of mutations and phosphosites found in hybrid clusters.

File Name: Supplementary Data 5.
Description: Hybrid clusters containing known activating mutations.

File Name: Supplementary Data 6.
Description: Hybrid clusters containing recurrent mutations.

File Name: Supplementary Data 7.
Description: TCGA RPPA analyses of differentially-expressed proteins harboring co-clustering mutations.

File Name: Supplementary Data 8.
Description: CPTAC MS proteomic data analyses of differentially-expressed proteins harboring co-clustering mutations.

File Name: Supplementary Data 9.
Description: Gene-level enrichment of co-clustered mutations tested to be functionally activating.

File Name: Supplementary Data 10.
Description: Associations of genetic dependency in cell lines with co-clustering mutations across tissue lineages in the DepMap dataset.

File Name: Supplementary Data 11.
Description: Co-clustering phosphosites found in CPTAC global phosphoproteomics data of primary tumors.

File Name: Supplementary Data 12.
Description: Literature review of co-clustering phosphosites implicated in cancer.
